# An observational study of the reliability and concurrent validity of heart rate variability devices in athletes

**DOI:** 10.3389/fphys.2025.1707318

**Published:** 2026-01-07

**Authors:** Hedvig Johansson, Emily Adderley, Seán Clarke, Patrick McIntyre, Garreth Reilly, Brian Caulfield, Sinead Holden

**Affiliations:** 1 School of Public Health, Physiotherapy and Sports Science, University College Dublin, Dublin, Ireland; 2 Insight Centre for Data Analytics, University College Dublin, Dublin, Ireland; 3 UCD Institute for Sport and Heat, University College Dublin, Dublin, Ireland

**Keywords:** athlete monitoring, heart rate variability, photoplethysmography, pNN50, reliability, RMSSD, validity

## Abstract

**Background:**

Heart rate variability (HRV) is a non-invasive indicator of autonomic nervous system function and is increasingly used in athlete monitoring. While electrocardiography (ECG) is the gold standard for HRV measurement, its use is limited in field settings.

**Objective:**

To evaluate the intra-session reliability and concurrent validity of a smartphone-based PPG app for HRV measurement in athletes, compared to a Polar H10 chest strap and ECG.

**Methods:**

This observational study included 37 trained participants (17 female; mean age 21.95 ± 3.69 years). HRV was recorded concurrently via ECG, Polar H10 chest strap, and the CameraHRV smartphone app in two repeated trials on the same day. Data were processed using device-specific software. Intra-class correlation coefficients (ICC), coefficient of variation (CV%), mean absolute percentage error (MAPE), and Bland–Altman plots were used to assess reliability and agreement.

**Results:**

All three devices showed good-to-excellent intra-session reliability for RMSSD (ICC range 0.83–0.90) and pNN50 (ICC range 0.87–0.92). The Polar chest strap had the highest consistency and lowest error compared to ECG (RMSSD MAPE: 2.16%). The PPG app also demonstrated strong validity (RMSSD MAPE: 17.49%) but wider limits of agreement.

**Conclusion:**

Both the Polar chest strap and smartphone PPG app demonstrated acceptable reliability and validity for short-duration HRV assessment in athletes. While the chest strap outperformed the PPG app in precision, the PPG app may offers a practical, low-cost alternative for athlete monitoring.

## Introduction

Heart rate variability (HRV) is defined as the variation in time intervals between consecutive heartbeats (R-R intervals) (Shaffer et al., 2014). It is recognised as a key physiological marker of autonomic nervous system function, reflecting the dynamic interplay between the sympathetic and parasympathetic branches. Under physiological stress (illness/disease, stress, or even intense training), the rhythm becomes less variable due to the decreased parasympathetic activity and increase sympathetic activity (Shaffer et al., 2014; Katona et al., 1982; Shaffer and Ginsberg, 2017).

As a result, HRV suppression is increasingly used to indicate physiological stress and has been associated with impaired adaptation, reduced performance, and early markers of overtraining in both endurance and strength athletes (Addleman et al., 2024; Altini and Amft, 2016; Piatri et al., 2021), and is used assess response to training (Addleman et al., 2024; Manresa-Rocamora et al., 2021; Bellenger et al., 2016). Although debate continues regarding the performance benefits of HRV-guided training (Carrasco-Poyatos et al., 2022), HRV- is increasingly being used in real-world athlete monitoring.

While electrocardiography (ECG) is the gold standard for HRV measurement (Shaffer and Ginsberg, 2017), it requires for specialised laboratory equipment, trained personnel, and a controlled testing environment making it impractical for routine, real-world monitoring in sport and exercise settings (Plews et al., 2017; Ritchie et al., 2016; Buchheit, 2015). The combination of ultra-short term recordings (<5 min) and wearable technologies and mobile sensors have become increasingly popular alternatives (Altini and Amft, 2016), with our recent review showing >200 mobile applications available on the apple and google stores to measure and/or provide feedback on HRV (de Jager, 2025). These apps are generally used to support decision-making by coaches and athletes (Addleman et al., 2024). However, questions remain regarding measurement accuracy, reliability, and inter-device consistency, especially in athletic populations.

Many wearable devices and mobile applications rely on photoplethysmography (PPG). PPG is a non-invasive optical technique that uses a light source and a photodetector to detect variations in blood volume producing a pulse waveform with each heartbeat). Nonetheless, PPG-based devices are susceptible factors such as motion artifacts, variability in finger placement, and light interference (Scardulla et al., 2023). Despite some studies have evaluated the inter-session test-retest reliability of PPG sensors, there is yet to be consensus on the intra session reliability.

The aim of this study was to evaluate the intra-session reliability and concurrent validity of a camera based PPG HRV measurement compared to a chest strap and the gold standard ECG in athletes.

## Methods

### Design

This study was designed as an observational study, assessing the intra-session reliability and concurrent validity of a camera-based PPG sensor for HRV. This was compared to the real-word reference standard, a chest strap, and three lead ECG. The reporting of this study follows the Guidelines for Reporting Reliability and Agreement Studies (GRRAS) (Kottner et al., 2011). Participants visited the lab on one occasion, where HRV was measured concurrently by three devices (as outlined below), repeated twice. The testing sessions were conducted between 8 a.m. and 3 p.m. at the UCD Institute for Sport and Health. Ethical approval was granted by the School Research Ethics Committee (UTMREC-24-106) at University College Dublin and performed in accordance with the principles of the Declaration of Helsinki. All participants provided written informed consent prior to data collection. All participants received an information sheet outlining the study’s procedures, potential risks, and benefits.

### Participants

A sample size calculation was conducted using the ICC.sample.size package in R, targeting an intraclass correlation coefficient (ICC) of 0.83 with a minimum acceptable threshold of 0.60, assuming two repeated measurements, a confidence level of 95%, and a power of 80%. This analysis indicated that a minimum of 34 participants would be required to detect reliable inter-session agreement.

Participants were recruited via flyers across the university campus, from local/university sports clubs, and through word-of-mouth. Participants were eligible for inclusion if they were 18 years of age or older, engaging in regular training at least three times per week, and classed as Tier two or above of the Participant Classification Framework (Shaffer and Ginsberg, 2017). Individuals were excluded if they had known autonomic, cardiovascular, or respiratory conditions or were taking medication that could affect heart rate or heart function.

### Procedures

After providing consent, anthropometric measurements including body mass and height were taken for each participant using a calibrated digital scale and wall-mounted stadiometer respectively. Participants then completed an online questionnaire on sport characteristics (primary sport, level of participation, training frequency), and self-reported stressors (caffeine, alcohol, smoking, sleep quality, sleep duration, and timing, duration and details of their last training session) (Addleman et al., 2024).

Subsequently, HRV measurements were taken in a light and temperature-controlled room which remained silent. Participants were in supine, reclined at a 45° reclined angle, with arms relaxed by their side, and were instructed to remain motionless. A 5-min rest period to allow for autonomic stabilisation, consistent with HRV measurement procedures was given prior to each recording. HRV metrics were recorded simultaneously for all three devices for a 60 s duration. The measurement protocol for each device is outlined below. During each test, participants were instructed to keep their eyes closed and breathe naturally without any imposed pace, consistent with best-practice guidelines in HRV methodology (Piatrikova et al., 2021). A 60-s recording duration was used for all HRV measurements in this study. This decision reflects the widespread adoption of short-duration recordings in commercial wearable devices and mobile health apps. Although longer recordings (e.g., 5 min) are considered the gold standard for HRV analysis, particularly for frequency-domain metrics and SDNN. Therefore, research is needed to establish the reliability and validity of these ultra-short-term recordings in real-world, ecological contexts, which was the aim of the current study.

To evaluate reliability, the entire procedure was repeated twice under the same conditions. After the first test, the ECG leads were removed, and the participants were allocated a standing time of 30 s. Following this period of standing, the participants conducted another 5-min seated rest period for autonomic recovery. This was followed by a second test under the same conditions, as outlined below.

The ECG was performed with a three-lead electrocardiogram, in which electrodes with conductive gel were placed at the following anatomical locations: left medial malleolus, right medial malleolus and right wrist (Application Note 109: 1-, 3-, 6- and 12-Lead ECG. BIOPAC Systems, Inc., 2019). Prior to electrode placement, the skin of each participant was prepared by shaving, abrading and cleaned. The Acknowledge software (v 4.4, BIOPAC, Goletta, CA, USA) was used to collect and process the ECG signal.

Participants were fitted with the Polar H10 chest strap, which was dampened with water to ensure the optimal conduction as per the manufacturer’s guidelines (Buchheit, 2015). The moistened elastic electrode strap was positioned at the xiphoid process level at a length which fitted the participants chest circumference (Buchheit, 2015). Data from the Polar chest strap was paired using Bluetooth Low Energy and processed via the Kubios HRV Scientific Lite 3.5.0 software (Kubios, LTD., Kuopio, Finland) which supports recording and processing of data for beat-to-beat RR interval data using the most common HRV indices (list).

**FIGURE 1:**
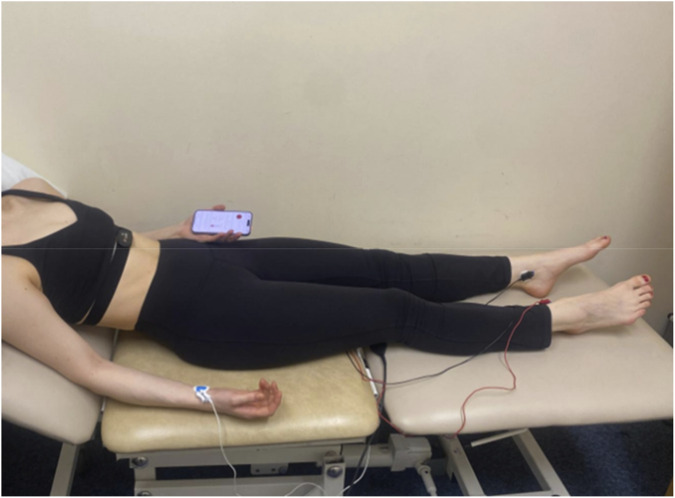
Test set-up of participant

PPG was measured using the CameraHRV app version 5.0.9 (A.S.M.A. B.V), operated on a compatible smartphone. The same smartphone was used for all measures. The app captures PPG using the camera at 30 frames per second, applying filtering techniques and using cubic spline interpolation to enhance the signal resolution to 180 HZ (Plews et al., 2017). Participants were instructed to lightly position their index finger over the camera lens, covering both the flash and camera as per the applications on screen guidance. The recordings were monitored on all three devices simultaneously (Figure 1).

### Data processing

Kubios HRV Scientific Lite 3.5.0 was used for HRV analysis for the Polar H10, while Camera HRV was used for the PPG. The ECG was processed using Acqknowledge Software.

The time-domain root mean of successive R-R interval differences (rMSSD) was used as the primary HRV calculation due to its widespread use in athlete monitoring and its robust correlation with parasympathetic activity. In addition to this the PNN50 were used as a secondary outcome. SDNN and heart rate were computed for the PPG and the chest strap only, therefore, the Polar chest strap was used as the reference device for these comparisons (Table 1). Prior to analysis data was visually inspected for normality.

**TABLE 1:** List of HR and HRV features, along with their definitions, used in the validation analysis.

Feature (units)	Definition	Devices
rMSSD (ms)	The square root of the mean of the sum of the squares of differences between adjacent intervals	PPG, heart rate monitor, ECG
pNN50	Proportion of successive normal heartbeats (NN) intervals that differ by more than 50 ms	PPG, heart rate monitor, ECG
Heart rate (bpm)	Number of beats per minute	PPG, heart rate monitor
SDNN	Standard deviation of the inter beat interval of normal beats	PPG, heart rate monitor

### Statistical analysis

All data were analysed using Studio. Descriptive statistics are presented as mean ± standard deviation (SD) unless otherwise stated. The normality of data distributions was assessed using histograms and QQ plots.

To evaluate the test–retest reliability, a two-way mixed-effects Intra-Class Correlation Coefficients (ICCs) with absolute agreement. ICC values were interpreted as follows: values below 0.5 indicated poor reliability, values between 0.5 and 0.75 indicated moderate reliability, values > 0.75 and 0.9 indicated good reliability, and values > 0.9 indicated excellent reliability.

Within-subject variability relative to the mean across tests was assessed using the Coefficient of Variation (CV%). A CV% of less than 10% was considered low and indicative of high consistency, a CV% between 10% and 20% was classified as moderate, and a CV% exceeding 20% was regarded as high, indicating greater variability.

Mean Absolute Percentage Error (MAPE) was calculated for each device compared to the ECG. Bland–Altman plots were used to evaluate agreement between devices (the PPG and chests strap) and the ECG.

## Results

### Participants

Thirty seven participants were included, the characteristics of which are detailed in Table 2. In addition, one person was removed from the reliability analysis due to missing repeat data leaving a total of 36 participants (17 females, 19 males) for the reliability analysis, and 37 for the validity. Of the 37 participants, two were elite/international athletes, 6 Highly Trained/National Level athletes and 29 were Trained/Developmental.

**TABLE 2:** Participant characteristics.

Characteristic	Total sample (n = 37)
Age (years)	21.95 ± 3.69
Sex (n)	Male (20)/Female (17)
Height (cm)	174.3 ± 9.02
Body Mass (kg)	73.38 ± 11.51
BMI (kg/m^2^)	24.6 ± 2.6

Data are presented as mean ± SD, or N.

### Reliability

Both the ECG and PPG measurements for RMSSD and pNN50 demonstrated good reliability (ICC >0.8; Table 3). In comparison, the Polar device exhibited excellent agreement for both metrics (ICC >0.9). All three devices demonstrated moderate variability in SD for RMSSD and pNN50, with the Polar chest strap exhibiting the most consistent results. This finding was supported by the CV% values, which indicated acceptable levels of reliability across all devices.

**TABLE 3:** Reliability statistics.

Device	Metric	Test 1 mean	Test 2 mean	CV%	ICC (95%)
ECG	RMSSD	57.9	58.1	14.22	0.88 (0.77, 0.93)
Chest strap	RMSSD	57	56.8	13.47	0.9 (0.82, 0.95)
PPG	RMSSD	60.3	60.2	14.6	0.83 (0.69, 0.91)
ECG	pNN50	30.5	31.8	N/A	0.89 (0.80, 0.94)
Chest strap	pNN50	29.7	31	N/A	0.92 (0.85, 0.96)
PPG	pNN50	32	33.7	N/A	0.87 (0.77, 0.93)

All pNN50 CV scores were reported as N/A due to zero values across participants, precluding CV calculation.

Abbreviations: SD, standard deviation; ICC, interclass correlation coefficient; ECG, electrocardiogram; PPG, photoplethysmography; RMSSD, root mean square of successive differences; pNN50, percentage of successive R-R intervals that differ by more than 50 ms.

### Validity

**FIGURE 2:**
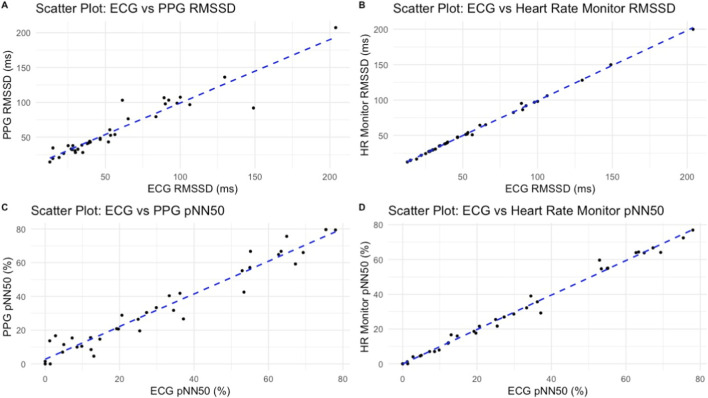
Scatter plot of successive R-R interval differences (RMSSD) for the PPG **(A)**and Heart rate monitor **(B)**compared to ECG, and the proportion of successive normal heartbeats (NN intervals) that differ by more than 50 ms (pNN50) for the PPG **(C)**and Heart rate monitor **(D)**compared to ECG. Comparisons between PPG and Polar chest strap for heart rate and SDNN are summarised in Table 5, with heart rate values showing high agreement and no significant difference between tools.

Validity outcomes comparing the Polar chest strap and PPG to ECG-derived HRV values (RMSSD and pNN50) are presented in Table 4; Figure 2. The Polar chest strap showed the closest agreement with ECG across both indices, while PPG demonstrated larger bias and greater variability.

**TABLE 4:** Comparison between devices and gold standard ECG for RMSSD and pNN50.

Metric	Device	Mean bias from ECG (±SD)	95% limits of agreement	Correlation coefficient	MAPE (%)
RMSSD	Chest strap	0.4 ± 1.9 ms	−3.3–4.0 ms	1.0	2.16
RMSSD	PPG	−3.0 ± 13.62 ms	−29.7–23.7 ms	0.95	17.49
pNN50	Chest strap	−0.4% ± 2.4%	−5.1%–4.4%	1.0	N/A
pNN50	PPG	1.9% ± 6.0%	−9.7%–13.4%	9.7	N/A

Abbreviations: MAPE, mean absolute percentage error; RMSSD, root mean square of successive differences; pNN50, percentage of successive R-R intervals that differ by more than 50 ms; PPG, photoplethysmography.

**TABLE 5:** Comparisons between PPG and polar chest strap.

Parameter	Chest strap mean ± SD	PPG mean ± SD	Bias (mean ± SD)	95% LoA	MAPE (%)
HR (bpm)	67.81 ± 11.62	68.22 ± 10.68	0.41 ± 3.68	−7.63, 6.81	10.62
SDNN (ms)	55.47 ± 30.81	63.11 ± 22.42	7.64 ± 17.43	−26.52, 41.81	60.9

Abbreviation: SD, standard deviation; PPG, photoplethysmography; LoA, limits of agreement; HR, heart rate; SDNN, standard deviation of the interval of normal sinus beats.

**FIGURE 3:**
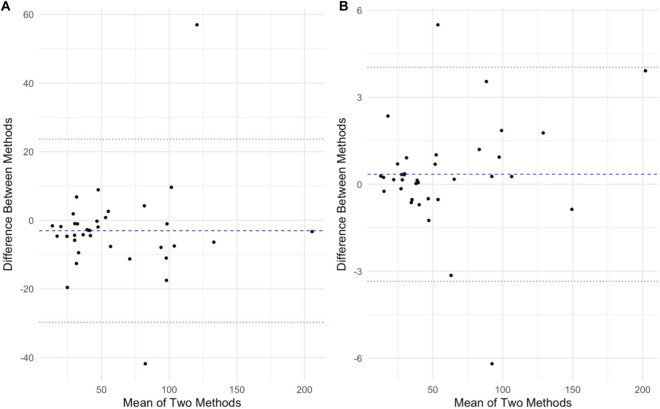
Bland-Altman for root mean of successive R-R interval differences (RMSSD) for the PPG **(A)** and Heart rate monitor **(B)** compared to ECG. Units are in ms. Blue line indicates mean difference (bias) and the red lines indicate upper and lower 95% limits of agreement.

**FIGURE 4:**
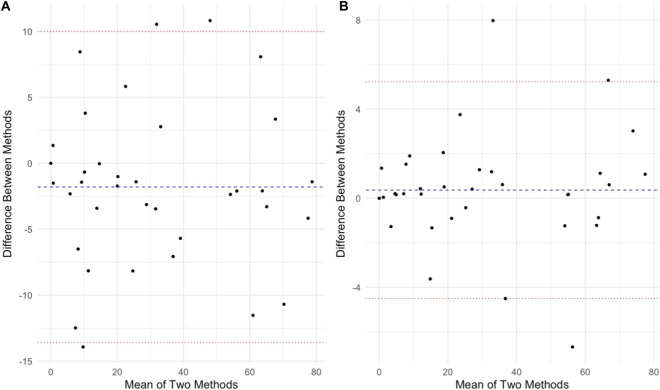
Bland-Altman for the proportion of successive normal heartbeats (NN intervals) that differ by more than 50 ms (pNN50) for the PPG **(A)**and Heart rate monitor **(B)**compared to ECG, data are presented as % values. Blue line indicates mean difference (bias) and the red lines indicate upper and lower 95% limits of agreement.

Figures 3, 4 show Bland-Altman plots illustrating agreement between each device and ECG for RMSSD and for pNN50, respectively. PPG exhibited greater limits for both outcomes (Table 4).

## Discussion

This study found that a smartphone-based photoplethysmography (PPG) app can reliably measure heart rate variability (HRV) in trained individuals, with results comparable to an ECG (electrocardiogram) under controlled, resting conditions. All three devices tested (ECG, Polar H10 chest strap, and smartphone PPG app) demonstrated good to excellent intra-session reliability for key time-domain HRV metrics (RMSSD and pNN50 ranging from 0.83 to 0.92), indicating that both the chest strap and the PPG app provided consistent measurements within a single session. Despite the positive results for the PPG, the chest strap outperformed the PPG measurement in almost all metrics with an almost perfect correlation with ECG, confirming its superior accuracy and consistency.

These results are consistent with previous literature, which has shown that both chest-strap HR monitors and PPG technologies can achieve high reliability under standardised conditions (Rogers et al., 2025). Plews et al. (2017) observed trivial differences in HRV assessed by RMSSD when comparing a smartphone PPG app (HRV4Training) and a Polar chest strap, and both methods correlated almost perfectly with the ECG (R ≈ 1.00). Similarly, Moya-Ramón et al. (2022) reported very strong to almost perfect correlations between smartphone-app HRV measurements and simultaneous ECG, with correlation coefficients ranging from r = 0.77 up to 0.94. These consistently high correlations indicate that, appropriate data acquisition and signal processing conditions, PPG apps can closely mirror ECG-derived HRV values.

In our data, the Polar device not only had nearly perfect agreement with ECG (bias and variability were minimal), but it even exhibited a slightly higher ICC for RMSSD than the ECG when comparing repeated measures. This was unexpected, given ECG is considered the gold standard for HRV. Supporting this, previous work has reported exceptionally high ICC agreement (>0.999) (Giles et al., 2016). One possible explanation is that subtle factors like electrode placement or signal noise can affect ECG consistency. Overall, this suggests the Polar H10 may serve as a valid alternative to ECG for estimating RMSSD, a key time-domain HRV metric.

The PPG app’s HRV readings were strongly correlated with the ECG values (Pearson r typically above 0.9 for RMSSD), showing that, on average, the app tracked fluctuations in heart rhythm very closely to the gold standard. The inherent sources of noise for a smartphone camera (like subtle finger movements or pressure changes) likely contributed to its CV of ∼14.6% in our data, but this level of variability is on par with the ECG’s variability here, emphasizing that PPG can perform comparably to ECG in a calm, controlled measurement scenario. Notably, even the Polar chest strap–arguably the most stable–showed a moderate CV in this study.

Interestingly, pNN50 values from CameraHRV showed slightly better agreement with ECG than RMSSD (r = 0.97; bias = 1.88 ± 5.95% versus r = 0.95; bias = −3.04 ± 13.62 ms). This pattern is somewhat surprising, as many previous studies have suggested RMSSD is the more robust and reliable time-domain metric, particularly for PPG-based measurements (Jeyhani et al., 2015). Jeyhani et al. found that PPG-derived HRV metrics showed only minimal errors relative to ECG for most indices, except pNN50 which showed a much larger error (∼30% discrepancy) (Jeyhani et al., 2015). This may be due to the short–term (60 s) nature of our measurement protocol. However, coefficient of variation (CV%) for pNN50 could not be computed. This may align with the known sensitivity of pNN50 to short measurement durations where there may not be any measures >50 ms, and underscores its limited stability in 60-s recordings (Shaffer and Ginsberg, 2017).

It is a practical concern is whether wearable devices and apps can capture short-term HRV fluctuations accurately. Some studies have highlighted discrepancies in HRV readings from wearables, even under controlled conditions, and particularly over very short recording durations (<5 min). Our data, using 1-min segments, show a strong alignment of the PPG and chest strap with the ECG for that ultra-short window. This builds on recent evidence that even ultra-short HRV measures (1 min) can be valid in athletes when using reliable devices (Moya-Ramon et al., 2022). The Bland–Altman analysis in our study (Figure 3) revealed that the PPG’s limits of agreement for RMSSD and pNN50 were wider than those of the chest strap vs. ECG. Readings from the PPG therefore could deviate from the ECG by a larger margin compared to the Polar’s deviations. Despite these limitations in absolute precision, the strong correlation and acceptable reliability of the PPG app highlight its practical value.

### Strengths and limitations

Our study extends the validation of wearable and app-based HRV tools specifically to an athletic population and within short recording durations. Most prior validation studies have focused on clinical or general populations, which can be a limitation because athletes often exhibit different cardio-autonomic profiles (e.g., higher vagal tone at rest, bradycardia, and cardiac remodelling) that might influence HRV readings.

Several limitations should be acknowledged when interpreting the findings of this study.

This investigation assessed intra-session reliability due to the known sensitivity of HRV to multiple physiological and environmental factors such as acute training fatigue. Given the athletic population’s variable training demands and routines, a single-session design was employed to minimise short-term influences, while still observing potential effects on validity. During data collection, participants were instructed to hold the smartphone in accordance with app instructions. This may have resulted in mild isometric contraction of the upper limb muscles. While this could introduce movement artefacts and influence the PPG signal, all devices were used simultaneously under the same conditions, meaning any such effect would have been consistent across devices. The primary HRV outcome measure in this study was RMSSD, a time-domain metric that reflects short-term parasympathetic activity and is less sensitive to respiratory rate and recording duration. Frequency-domain parameters (e.g., LF, HF, LF/HF) were not included, as their accurate interpretation typically requires recordings of at least 5 min in duration (Shaffer and Ginsberg, 2017).

### Practical applications

Both the Polar chest strap and PPG device demonstrated moderate to excellent reliability and validity in measuring HRV in trained individuals. While the chest strap outperformed PPG, PPG usage offers significant advantages in terms of accessibility and affordability, in this case available as a smartphone app. This makes them a practical option for users without specialised equipment, allowing for widespread and cost-effective HRV monitoring. Overall, these monitors may provide opportunity for trained individuals to use in their assessment of HRV within their training contexts.

## Conclusion

In summary, both the Polar chest strap and the PPG application showed acceptable intra-session reliability and validity compared to ECG for measuring RMSSD and pNN50. However, the Polar chest strap provided superior accuracy and lower variability. The choice between devices may thus be guided more by practical considerations, such as ease of use, cost and context of application. While PPG may offer a cheaper and more user-friendly alternative, it introduces greater measurement error, which may limit its application in elite sport settings where precision is critical.

## Data Availability

The raw data supporting the conclusions of this article will be made available by the authors, without undue reservation.
